# The novel IκB kinase β inhibitor IMD-0560 prevents bone invasion by oral squamous cell carcinoma

**DOI:** 10.18632/oncotarget.2640

**Published:** 2014-10-28

**Authors:** Yukiyo Tada, Shoichiro Kokabu, Goro Sugiyama, Chihiro Nakatomi, Kazuhiro Aoki, Hidefumi Fukushima, Kenji Osawa, Yasutaka Sugamori, Keiichi Ohya, Masato Okamoto, Tomoyuki Fujikawa, Akiko Itai, Kou Matsuo, Seiji Watanabe, Eijiro Jimi

**Affiliations:** ^1^ Division of Molecular Signaling and Biochemistry, Department of Health Promotion, Kyushu Dental University, Manazuru, Kokurakita-ku, Kitakyushu, Fukuoka, Japan; ^2^ Division of Dental Anesthesiology, Department of Control of Physical Functions, Kyushu Dental University, Manazuru, Kokurakita-ku, Kitakyushu, Fukuoka, Japan; ^3^ Section of Pharmacology, Department of Bio-Matrix, Graduate School, Tokyo Medical and Dental University, Yushima, Bunkyo-ku, Tokyo, Japan; ^4^ Department of Physiological Sciences and Molecular Biology, Fukuoka Dental College, Fukuoka, Tamura, Sawara-ku, Fukuoka, Japan; ^5^ Division of Pathophysiology, Research Center for Genomic Medicine, Saitama Medical University, Yamane, Hidaka-shi, Saitama, Japan; ^6^ Department of Advanced Immunotherapeutics, Kitasato University School of Pharmacy, Shirokane, Minato-ku, Tokyo, Japan; ^7^ Institute of Medical Molecular Design Inc (IMMD Inc), Hongo, Bunkyo-ku, Tokyo, Japan; ^8^ Division of Oral Pathology, Department of Health Promotion, Kyushu Dental University, Manazuru, Kokurakita-ku, Kitakyushu, Fukuoka, Japan; ^9^ Center for Oral Biological Research, Kyushu Dental University, Manazuru, Kokurakita-ku, Kitakyushu, Fukuoka, Japan

**Keywords:** NF-κB, IMD-0560, bone invasion, oral squamous cell carcinoma

## Abstract

Oral squamous cell carcinoma (OSCC) cells display significantly augmented nuclear factor-κB (NF-κB) activity, and inhibiting this activity suppresses malignant tumor characteristics. Thus, we evaluated the effect of IMD-0560, a novel inhibitor of IκB kinase (IKK) β that is under assessment in a clinical trial of rheumatoid arthritis, on bone invasion by the mouse OSCC cell line SCCVII. We examined the inhibitory effects of IMD-0560 on NF-κB activity and tumor invasion using human OSCC cell lines and SCCVII cells *in vitro*. Using a mouse model of jaw bone invasion by SCCVII cells, we assessed the inhibitory effect of IMD-0560 on jaw bone invasion, tumor growth, and matrix degradation *in vivo*. IMD-0560 suppressed the nuclear translocation of NF-κB and the degradation of IκBα in OSCC cells. IMD-0560 also inhibited invasion by suppressing matrix metalloproteinase-9 (MMP-9) production in OSCC cells. IMD-0560 protected against zygoma and mandible destruction by SCCVII cells, reduced the number of osteoclasts by inhibiting receptor activator of NF-κB ligand (RANKL) expression in osteoblastic cells and SCCVII cells, increased SCCVII cell death and suppressed cell proliferation and MMP-9 production in SCCVII cells. Based on these results, IMD-0560 may represent a new therapeutic agent for bone invasion by OSCC cells.

## INTRODUCTION

Oral squamous cell carcinoma (OSCC) is the most common malignant tumor of the oral cavity, head and neck [1-3]. The annual incidence of OSCC is estimated to be 400,000 to 500,000 new cases worldwide, which is predicted to increase in the next few decades. Curable lesions that are detected early are rarely symptomatic; thus, preventing fatal disease requires early detection by screening. Treatment consists of surgery, radiation, or both, although surgery plays a larger role in the treatment of most oral cavity cancer. The overall 5-year survival rate is > 50%. However, the poor prognosis for OSCC reflects a limited understanding of the mechanisms underlying local and regional invasion and metastasis that occur in a significant portion of patients, as well as the unsatisfactory responsiveness to conventional systemic therapy in recurrent and advanced disease.

Gingival squamous cell carcinomas frequently invade mandible bone, which is associated with worse prognosis and should be treated surgically by resection. The prevalence of mandibular bone involvement ranges from 12 to 56% [4-6]. The treatment results of these lesions are typically poor, with nearly 70% of cases recurring at the primary lesion site, ultimately causing death [7,8]. Although controversial, the bone destruction that occurs due to OSCC invasion is thought to be mediated by osteoclasts rather than the carcinoma itself [9]. Recent studies have established that bone resorption by osteoclasts is an important step in the process of bone invasion and metastasis in several types of malignancy, indicating that a complete understanding of the regulation of osteoclastogenesis by OSCC cells is necessary to prevent bone invasion by OSCC cells [9,10].

Nuclear factor-κB (NF-κB) represents a family of dimeric transcription factors that are contain a characteristic Rel homology domain. Latent NF-κB, which forms a complex with IκB, resides in the cytoplasm. This pathway can be rapidly and transiently activated by a wide variety of substances, such as mitogens, cytokines, and microbial components. NF-κB activation is dependent on a specific IκB kinase (IKK) complex composed of two catalytic subunits, IKKα (IKK1) and IKKβ (IKK2), and the regulatory subunit NF-κB essential modulator (NEMO/IKKγ). Upon activation, IKK phosphorylates specific serine residues in IκB proteins, triggering their ubiquitination and degradation by the proteasome, thus allowing the NF-κB dimers to translocate to the nucleus to regulate gene expression [11,12]. Gene-targeting experiments revealed that many pro-inflammatory stimuli require the IKKβ subunit for NF-κB activation. The NF-κB activation pathway contributes to the induction of inflammatory mediators, cytokines, chemokines, proteases and inhibitors of apoptosis, and it has been proposed that the NF-κB pathway might link inflammation to tumor promotion and progression [11,12].

The NF-κB signaling pathway is activated in many cancers, including OSCC, contributing to the acquisition of malignant characteristics, such as increased invasion, survival, chemoresistance, and angiogenesis of OSCC [13-15]. A previous report showed that elevated expression of NF-κB correlates to enhanced invasion and metastasis of OSCC [15]. Furthermore, the osteoclast differentiation factor receptor activator of NF-κB ligand (RANKL) activates NF-κB in osteoclast precursors to cause differentiation into osteoclasts, and selective inhibitors of NF-κB inhibit RANKL-induced osteoclastogenesis *in vitro* and *in vivo* [16-18].

Recently, novel synthesized chemical compounds that act as IKK inhibitors have been developed [19, 20]. We previously reported that a selective inhibitor of NF-κB, NBD peptide, which disrupts the association of NEMO with both IKKs, prevents bone invasion in an *in vivo* OSCC model [21]. However, it is very difficult to use this laboratory reagent for clinical application.

IMD-0560, or N-[2,5-bis(trifluoromethyl)phenyl]-5-bromo-2-hydroxybenzamide, was developed as novel inhibitor of IKK [22-24]. The molecular structure of IMD-0560 was designed by analyzing a binding mode of aspirin to IKKβ [19, 20]. This drug is a selective IKKβ inhibitor, blocks IκBα phosphorylation, and prevents NF-κB p65 nuclear translocation, and its prodrug is under assessment in a clinical trial for inflammation-related cardiovascular diseases and rheumatoid arthritis [19, 20]. In this study, we examined the potential for the clinical evaluation of IMD-0560 for the treatment of bone invasion by OSCC cells.

## RESULTS

### IMD-0560 inhibits TNFα-induced p65 phosphorylation and IκBα degradation in human and mouse OSCC cells

**Figure 1:**
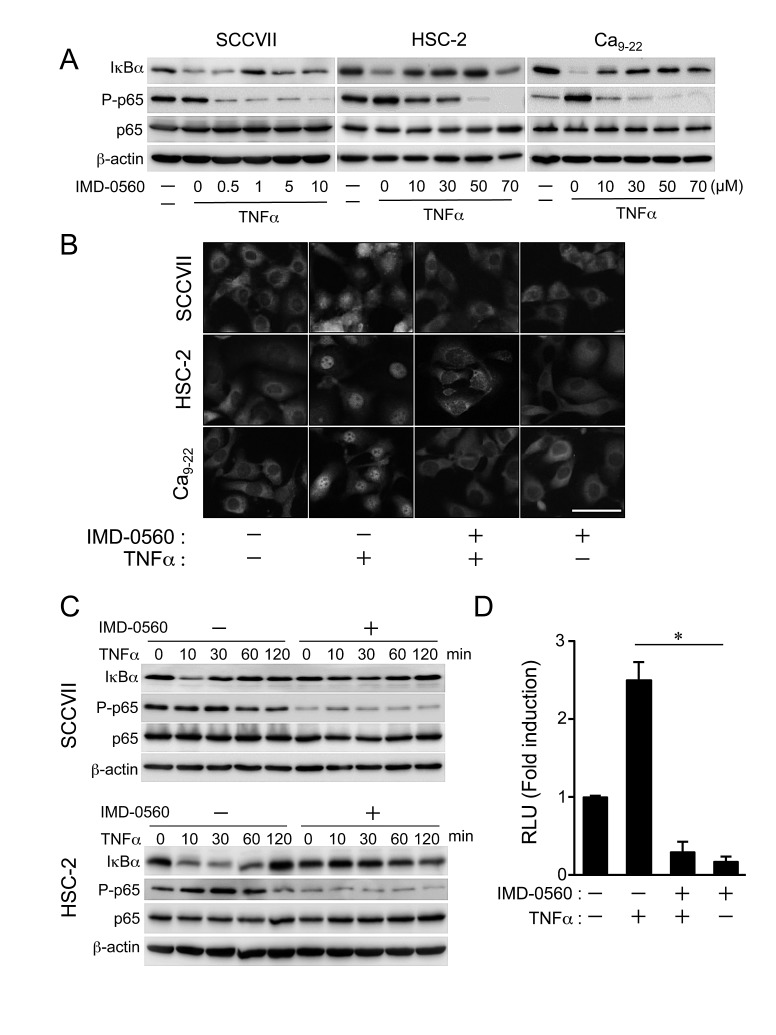
IMD-0560 inhibits TNFα-induced p65 phosphorylation and IκBα degradation in OSCC cells (A) SCCVII, HSC-2 and Ca_9-22_ cells were pretreated with various concentrations of IMD-0560 for 120 min and then treated with TNFα (10 ng/ml) for 15 min. p65 phosphorylation and IκBα degradation were examined via Western blot. β-actin was used as a loading control. Similar results were obtained in three independent experiments. (B) SCCVII, HSC-2 and Ca_9-22_ cells were pretreated with or without IMD-0560 (1 or 10 μM) for 120 min and then further treated with or without TNFα (10 ng/ml) for 30 min. Then, the cells were fixed and incubated in an anti-p65 antibody, followed by incubation in Alexa Fluor 430-conjugated anti-rabbit IgG. The subcellular localization of Alexa Fluor 430-labeled p65 was determined via fluorescence microscopy (magnification 200x). Bar = 50 μm. Similar results were obtained in three independent experiments. (C) SCCVII and HSC-2 cells were pretreated or without IMD-0560 (1 or 10 μM) for 120 min and then treated with TNFα (10 ng/ml) for the indicated periods. p65 phosphorylation and IκBα degradation were examined via Western blot. β-actin was used as a loading control. Similar results were obtained in three independent experiments. (D) SCCVII cells were transiently transfected with a PBIIx reporter, pretreated with or without IMD-0560 (1 μM) for 120 min and then treated with or without TNFα (10 ng/ml) for 8 hrs. The cells were assessed for luciferase activity after 8 hrs. The data are expressed as the mean ± SD (n=3). *p<0.01. Similar results were obtained in three independent experiments.

Pretreatment with IMD-0560 inhibited TNFα-induced p65 phosphorylation (Ser-536) and IκBα degradation in a dose-dependent manner in SCCVII, HSC-2, and Ca_9-22_ cells (Figure 1A). We used IMD-0560 at 1 μM for SCCVII and 10 μM for HSC-2 and Ca_9-22_ cells following experiments, respectively. TNFα induced the translocation of p65 from the cytoplasm to the nucleus, and IMD-0560 significantly blocked this translocation in HSC-2, Ca_9-22_, and SCCVII cells (Figure 1B). Pretreatment with IMD-0560 inhibited both IκBα degradation and p65 phosphorylation induced by TNFα (Figure 1C). IMD-0560 also suppressed TNFα-induced transcriptional activity (Figure 1D). These results strongly indicate that IND-0560 inhibits TNFα-induced NF-κB activation in OSCC cells.

### Inhibitory effect of IMD-0560 on the invasion of OSCC cells

**Figure 2:**
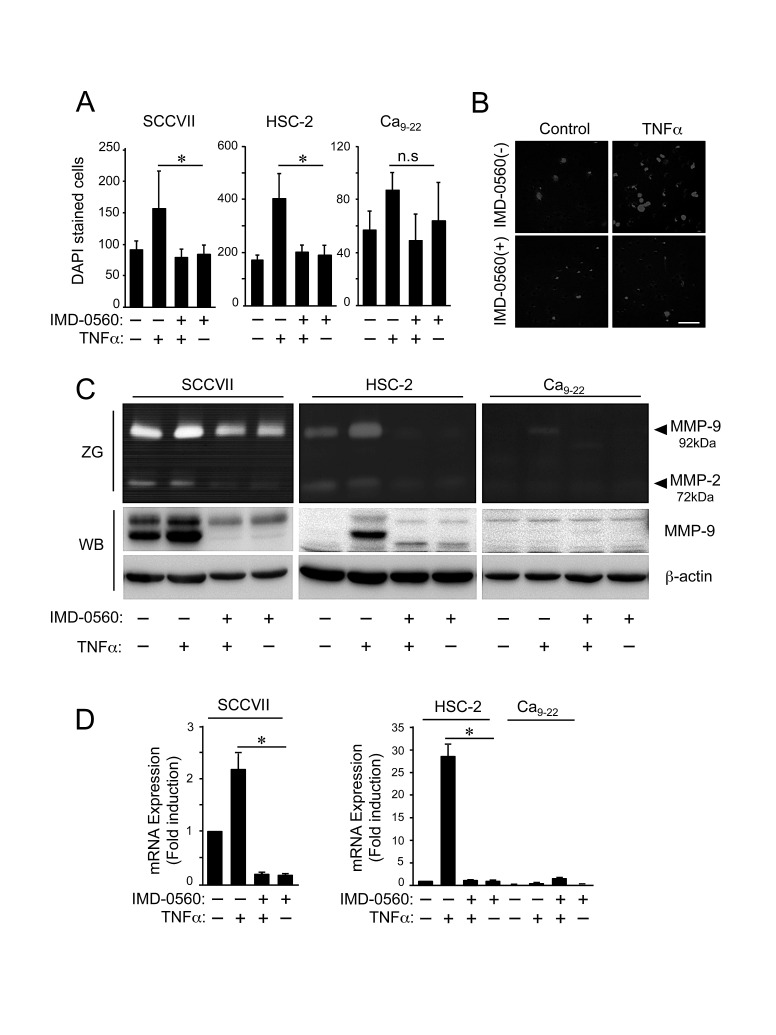
IMD-0560 inhibits cell invasion and MMP9 activity in OSCC cells (A) The cells suspended in serum-free DMEM were seeded in the upper chamber above a gelatin-coated porous membrane. IMD-0560 (1 or 10 μM) was placed in the upper chamber 2 hrs prior to TNFα treatment, and 10 ng/ml TNFα was placed in the lower chamber, followed by incubation for 24 hrs. Then, the cells attached to the upper surface of the membrane were scraped off, and the cells that migrated to the lower surface were fixed and stained with DAPI and then quantified. The data are expressed as the mean ± SD (n=3). *p<0.01. Similar results were obtained in three independent experiments. (B) A representative image of each group in SCCVII cells is shown (magnification 200x). Bar = 50 μm. Similar results were obtained in three independent experiments. (C) The cells were incubated in serum-free DMEM for 24 hrs in the presence or absence of TNFα pretreated with or without IMD-0560 (1 or 10 μM). The conditioned media were analyzed via gelatin zymography (ZG). The identical conditioned media were used for Western blot analysis (WB) to detect MMP-9. (D) The cells were pretreated with or without IMD-0560 (1 or 10 μM), followed by treatment with or without TNFα. Total RNA was isolated, and the *mmp-9* and *β-actin* mRNA levels were analyzed via real-time PCR. The data represent the mean ± SD of the expression levels of *mmp-9* relative to *β-actin* (n=3). *p<0.01. Similar results were obtained in three independent experiments.

TNFα markedly enhanced the invasion of SCCVII and HSC-2 cells but not Ca_9-22_ cells (Figures 2A and 2B). Pretreatment with IMD-0560 strongly inhibited TNFα-induced cell invasion, suggesting that NF-κB activation plays a role in OSCC invasion. The viability of the cells treated with IMD-0560 for up to 24 hrs was compatible with that of untreated cells (Supplementary Figure S1).

TNFα-induced cell invasion across a gelatin-coated membrane may partially occur due to the activity of gelatinase released from OSCC cells. Therefore, we investigated the effects of IMD-0560 on the production of 2 major gelatinases, MMP-2 and MMP-9, via gelatin zymography. TNFα induced 92 kDa gelatinase activity in SCCVII and HSC-2 cells, and this 92 kDa gelatinase corresponded to MMP-9; however, TNFα did not affect the release of MMP-2 (Figure 2C). Pretreatment with IMD-0560 suppressed MMP-9 activity by inhibiting MMP-9 expression (Figure 2D). Although TNFα slightly induced MMP-9 activity, but not MMP-2 activity in SCCVII and HSC-2 cells, TNFα failed to induce MMP-9 expression in Ca_9-22_ cells (Figure 2D). TNFα strongly increased the mRNA level of MMP-9, and IMD-0560 inhibited the TNFα-induced increase in MMP-9 mRNA expression.

### Early treatment with IMD-0560 inhibited bone invasion by SCCVII cells

**Figure 3:**
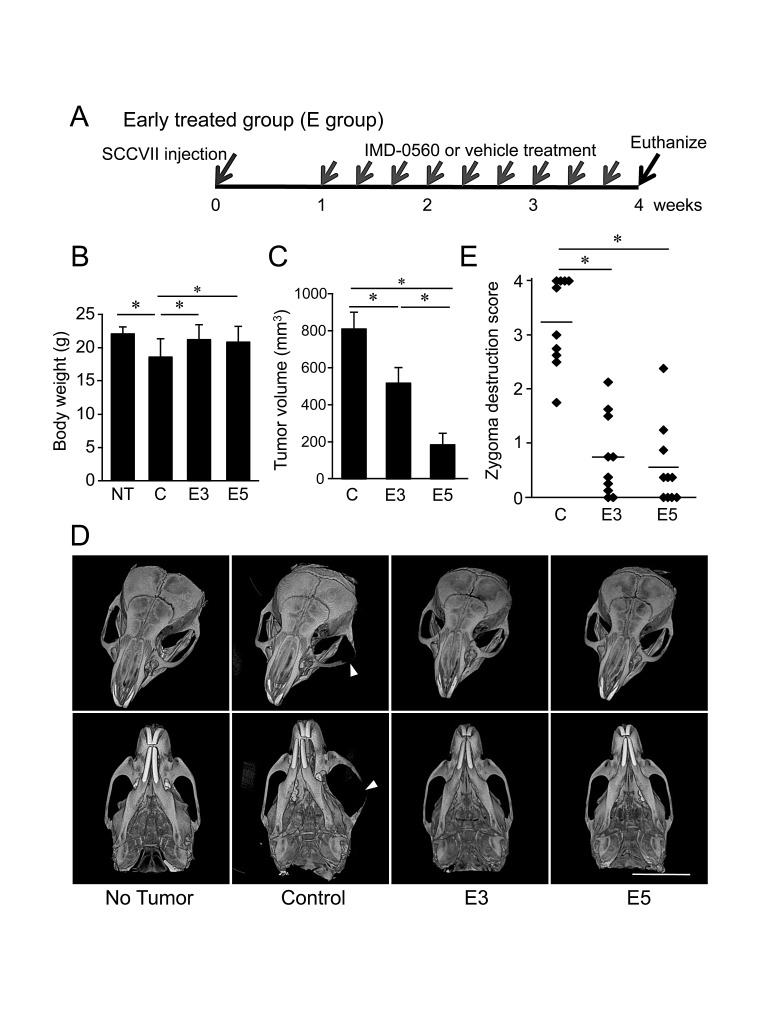
Early treatment with IMD-0560 inhibited bone invasion by SCCVII cells SCCVII cells were injected into the left masseter region of 8- to 10-week-old male C3H/HeN mice. One week after injection, the mice were locally treated with vehicle (50 μl of CMC: control, n=10) or IMD-0560 (3 or 5 mg/kg in 50 μl of CMC, n=10 each) 3 times per week for 3 weeks. (A) Protocol of IMD-0560 treatment. (B) In the SCCVII cell mouse model, the body weights were measured 28 days after tumor inoculation. Mice treated with CMC alone served as controls. NT: no tumor inoculation, C: control, E3: mice treated with IMD-0560 at 3 mg/kg, E5: mice treated with IMD-0560 at 5 mg/kg. (C) The tumor volume was assessed using calipers and was calculated using the following formula: width^2^ × length × 0.52. The data represent the mean ± SD; *p<0.05. (D) Reconstructed μ-CT images of zygoma destruction in the control and IMD-0560-treated mice. The arrowheads indicate destruction of the zygoma, and the arrow indicates a fracture line. Bar: 8 mm. (E) The severity of zygoma destruction was assessed based on clinical scoring of the control and IMD-0560-treated mice. *p<0.01.

To examine the inhibitory effects of IMD-0560 on bone invasion by SCCVII cells using mouse bone invasion model, we first treated the animals with IMD-0560 3 times per week for 3 weeks beginning 1 week after SCCVII injection (Figure 3A). Although the average body weight was slightly reduced in the control group, it was unchanged in the IMD-0560-treated groups compared with those of the no injection group (Figure 3B). The tumor size was reduced in the IMD-0560-treated groups in a dose-dependent manner, and no tumor was detected in some mice treated with 5 mg/kg IMD-0560 (Figure 3C).

The μ-CT images revealed the typical extent of bone invasion in all groups (Figure 3D). Figure 3E shows the zygomatic bone destruction score. In the control group, the zygoma and the mandible were severely destroyed (Figures 3D and 3E). In some IMD-0560-treated mice, we observed fractures of the zygoma. However, zygoma destruction was significantly suppressed in the IMD-0560-treated groups compared to the control groups. Although the tumor size in the 5 mg/kg IMD-0560-treated group was smaller than that of the 3 mg/kg IMD-0560-treated group, the inhibitory effect of each treatment on zygoma destruction was similar (Figures 3D and 3E).

**Figure 4:**
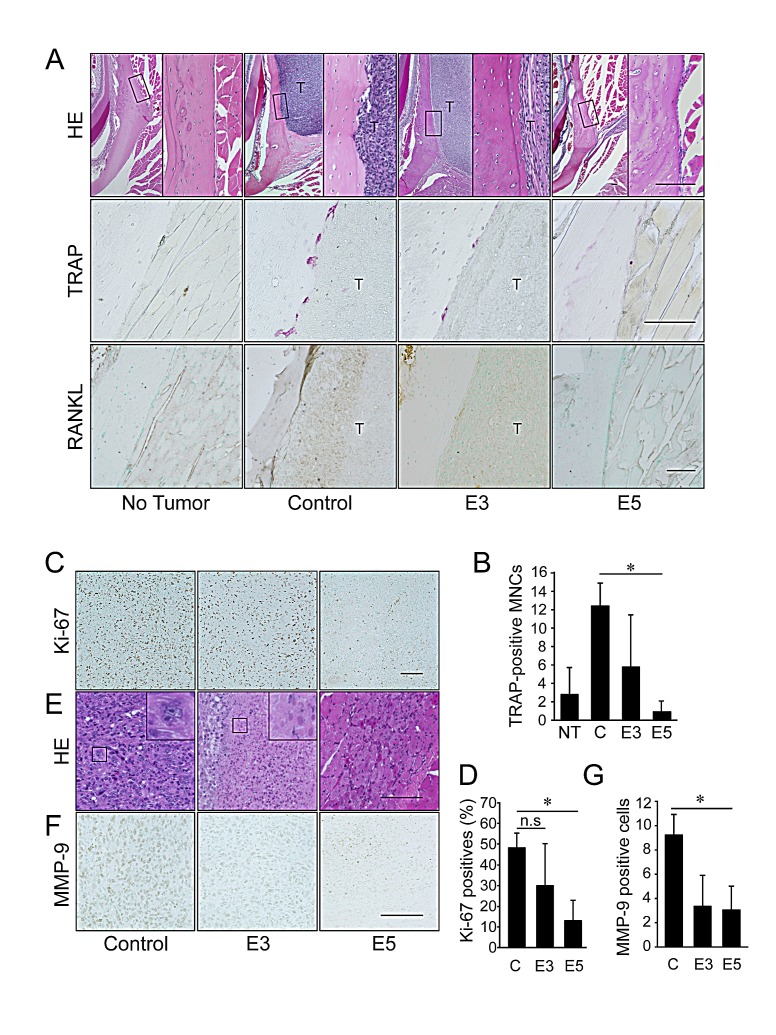
Early treatment with IMD-0560 reduced bone invasion by inhibiting osteoclastogenesis Twenty-eight days after tumor inoculation, the tissues were fixed in 3.7% formaldehyde, decalcified in 10% EDTA, sectioned in the coronal axis and stained with H&E (upper panels), TRAP (middle panels) or RANKL (lower panels). Mice treated with CMC alone served as controls. NT: no tumor inoculation, C: control, E3: mice treated with IMD-0560 at 3 mg/kg, E5: mice treated with IMD-0560 at 5 mg/kg. (A) Upper panels: Original magnification 40x. Bar=100 μm. Middle panels: Original magnification 400x. Bar=100 μm. Lower panels: Some specimens from each group were processed for immunohistochemical staining with an anti-RANKL antibody. Original magnification 200x. T: tumor. Bar=100 μm. Mice treated with CMC alone served as controls. (B) In each specimen, 5 tumor fields were randomly selected, and the number of TRAP^+^ MNCs was counted. The data are expressed as the mean ± SD of the number of TRAP^+^ MNCs/bone surface (mm^2^)/section (n=10). *p<0.05. Similar results were obtained in three independent experiments. (C) A representative photograph of Ki-67 staining in a tumor from the control and IMD-0560-treated groups *in vivo*. Ki-67-positive SCCVII cells were clearly identifiable based on brown staining (magnification 200x). Bar=100 μm. (D) The number of Ki-67-positive cells was counted in 5 randomly selected fields of each specimen. The data are expressed as the numbers of Ki-67-positive cells/total number of tumor cells/field. *p<0.05. (E) A representative photograph of H&E staining in the tumor of control and IMD-0560-treated groups *in vivo* (magnification 400x). Bar=100 μm. (F) A representative photograph of MMP-9 staining of a tumor from the control and IMD-0560-treated groups *in vivo*. MMP-9-positive SCCVII cells were clearly identifiable based on brown staining (magnification 400x). Bar=100 μm. (G) The number of MMP-9-positive cells were counted in 5 randomly selected fields of each tumor specimen. The data are expressed as the percentage of MMP-9-positive cells/total number of tumor cells/field. *p<0.01.

Histological analysis revealed resorption of the mandibular bone, including irregularities in the bone surface and an area of osteoclast occupation at the tumor/bone interface, in the control group (Figure 4A, upper and middle panels). The tumors were barely detected in the 5 mg/kg IMD-0560-treated group (Figure 4A). Many osteoclasts were easily identified in the control group after TRAP staining (Figures 4A, middle panels, and 4B). A few osteoclasts were detected in the 3 mg/kg IMD-0560-treated group, and none were detected in the 5 mg/kg IMD-0560-treated group (Figures 4A, middle panels, and 4B). In addition to the SCCVII cells that express RANKL, RANKL-positive osteoblastic cells were located at the interface between the bone and the SCCVII cells, presumably reflecting an increase in the number of osteoclasts in the control group (Figure 4A, bottom panels). RANKL expression in both the SCCVII cells and the osteoblastic cells was reduced in the 3 mg/kg IMD-0560-treated group (Figure 4A, bottom panels). No RANKL-positive cells were detected near the bone surface in the 5 mg/kg IMD-0560-treated group. Furthermore, IMD-0560 suppressed RANKL expression in osteoblasts and RANKL-induced osteoclastogenesis *in vitro* (Supplementary Figure S2).

The tumor size in the IMD-0560-treated mice was decreased (Figure 3C), suggesting that IMD-0560 treatment inhibited cell proliferation and/or cell death compared to the control treatment. Immunoreactivity for Ki-67, a marker of proliferation, was localized to the cell nuclei. IMD-0560 treatment significantly reduced the number of Ki-67-positive SCCVII cells compared to the control treatment (Figures 4C and 4D), suggesting that IMD-0560 treatment inhibited tumor cell proliferation. IMD-0560 suppressed cell proliferation, suggesting that the IMD-0560-induced inhibition of NF-κB directly suppressed the proliferation of SCCVII cells (Supplementary Figure S3). Next, we examined whether IMD-0560 treatment increased SCCVII cell death *in vivo*. Most tumor cells were intact in the control group, whereas in the 3 mg/kg IMD-0560-treated group, some foci of necrotic cells, characterized by an eosinophilic cytoplasm and nuclear changes such as pyknosis, karyorrhexis and karyolysis, were detected. The number of TUNEL-positive apoptotic cells was increased in the 3 mg/kg IMD-0560-treated group (Supplementary Figure S4). Carcinoma cells were nearly absent from the site of cell injection in the 5 mg/kg IMD-0560-treated group (Figure 4E). Furthermore, we assessed the number of apoptotic cells using Annexin V and PI staining *in vitro*. IMD-0560 induced apoptosis, suggesting that the IMD-0560-induced inhibition of NF-κB directly activated apoptosis in SCCVII cells (Supplementary Figure S1). IMD-0560 treatment also reduced the number of MMP-9-positive SCCVII cells (Figures 4F and 4G).

### IMD-0560 injection was useful for the treatment of bone invasion by SCCVII cells

**Figure 5:**
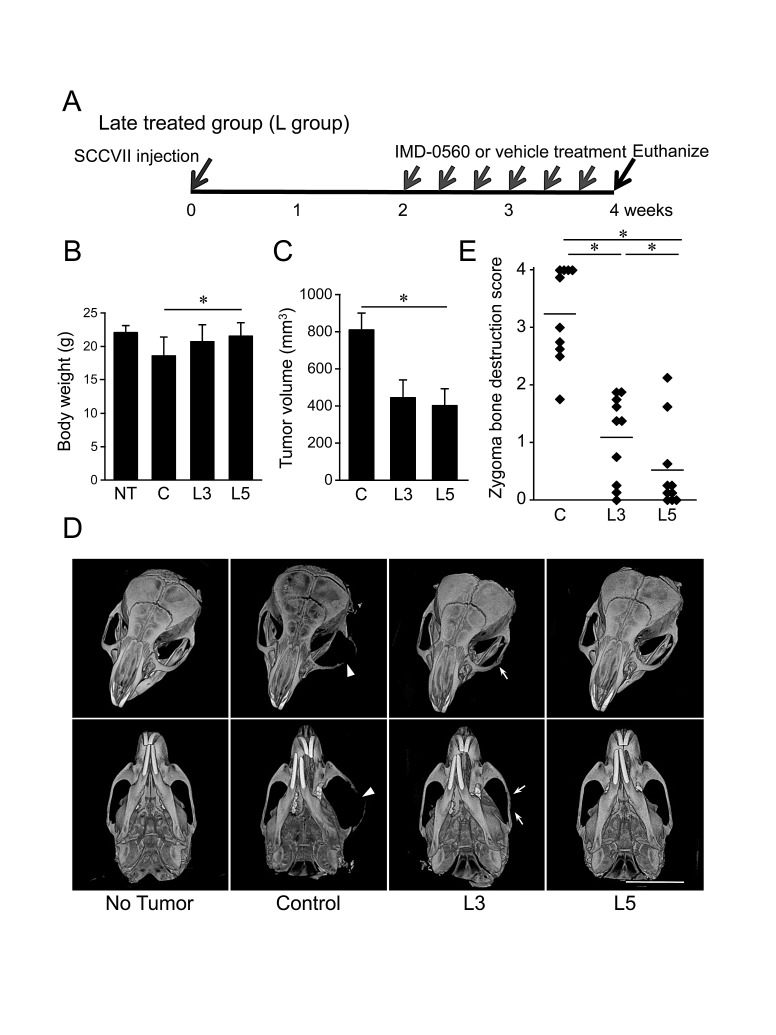
IMD-0560 injection was useful for the treatment of bone invasion by SCCVII cells SCCVII cells were injected into the left masseter region of 8- to 10-week-old male C3H/HeN mice. Two weeks after injection, the mice were treated locally with vehicle (50 μl of CMC, n=10) or IMD-0560 (3 or 5 mg/kg in 50 μl of CMC, n=10) 3 times per week for 2 weeks. (A) Protocol of IMD-0560 treatment. (B) In the SCCVII cell mouse model, the body weights were measured 28 days after tumor inoculation. Mice treated with CMC alone served as controls. NT: no tumor inoculation, C: control, L3: mice treated with IMD-0560 at 3 mg/kg, L5: mice treated with IMD-0560 at 5 mg/kg. (C) The tumor volume was assessed using calipers and was calculated using the following formula: width^2^ × length × 0.52. Mice treated with PBS served as controls. The data represent the mean ± SD; *p<0.05. (D) Reconstructed μCT images of zygoma destruction in the control and IMD-0560-treated mice. The arrowheads indicate destruction of the zygoma, and the arrow indicates a fracture line. Bar: 8 mm. (E) The severity of zygoma destruction was assessed based on clinical scoring of the control and IMD-0560-treated mice. *p<0.05.

To further examine whether IMD-0560 local injection is useful for the treatment of bone invasion, we treated animals with IMD-0560 3 times per week for 2 weeks beginning 2 weeks after SCCVII injection (Figure 5A). Before IMD-0560 injection, we detected tumor growth in all mice. Although the average body weight was slightly reduced in the control group, it was unchanged in the IMD-0560-treated groups compared with the no injection group (Figure 5B). The tumor size was reduced by up to half in the IMD-0560-treated groups, but there was no difference between the 3 mg/kg and 5 mg/kg IMD-0560-treated groups (Figure 5C).

The μ-CT images revealed severe destruction of the zygoma and the mandible in the control mice (Figure 5D). In some 3 mg/kg IMD-0560-treated mice, we detected fractures of the zygoma. However, zygoma destruction was significantly suppressed in the IMD-0560-treated groups compared to the control groups. Treatment with 5 mg/kg IMD-0560 prevented zygoma destruction more effectively than treatment with 3 mg/kg IMD-0560 (Figures 5D and 5E).

**Figure 6:**
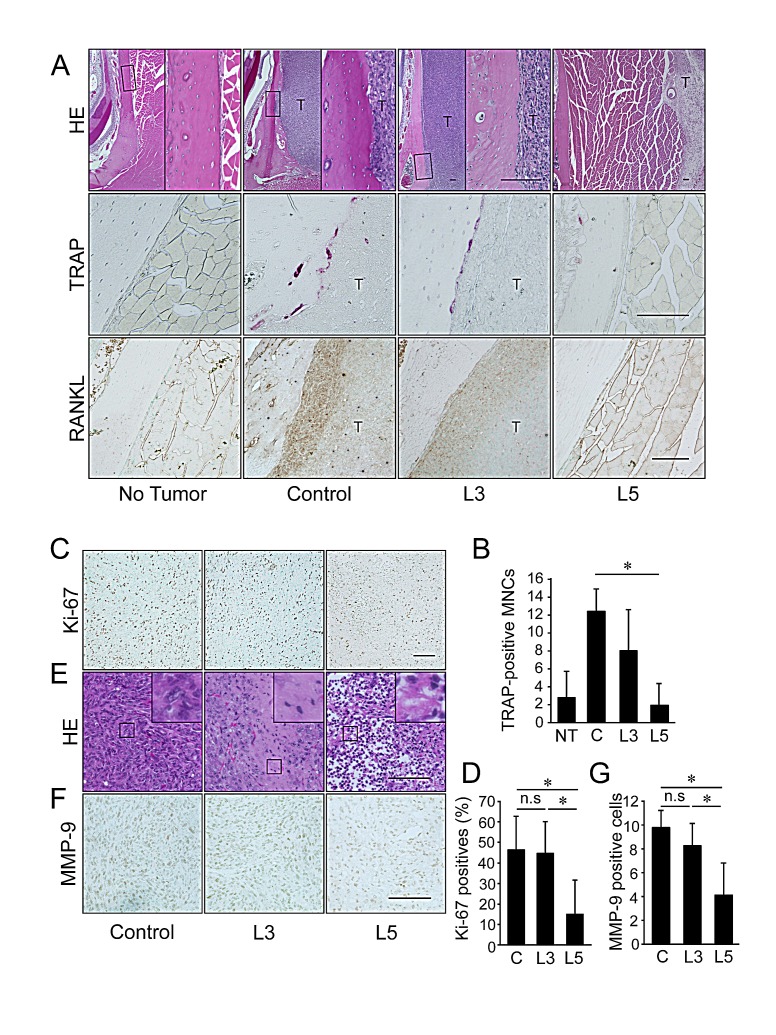
IMD-0560 injection was useful for the treatment of bone invasion by SCCVII cells via the inhibition of osteoclastogenesis Twenty-eight days after tumor inoculation, the tissues were fixed in 3.7% formaldehyde, decalcified in 10% EDTA, sectioned in the facial axis and stained with H&E (upper panels), TRAP (middle panels) or RANKL (lower panels). Mice treated with CMC alone served as controls. NT: no tumor inoculation, C: control, L3: mice treated with IMD-0560 at 3 mg/kg, L5: mice treated with IMD-0560 at 5 mg/kg. (A) Upper panels: Original magnification 40x. Bar=100 μm. Middle panels: Original magnification 400x. Bar=100 μm. Lower panels: Some specimens from each group were processed for immunohistochemical staining with an anti-RANKL antibody. Original magnification 200x. T: tumor. Bar=100 μm. (B): In each specimen, 5 tumor fields were randomly selected, and the number of TRAP^+^ MNCs was counted. The data are expressed as the mean ± SD of the number of TRAP^+^ MNCs/bone surface (mm^2^)/section (n=10). *p<0.05. Similar results were obtained in three independent experiments. (C): A representative photograph of Ki-67 staining in a tumor from the control and IMD-0560-treated groups *in vivo*. Ki-67-positive SCCVII cells were clearly identifiable based on brown staining (magnification 200x). Bar=100 μm. (D) The number of Ki-67-positive cells were counted in 5 randomly selected fields of each specimen. The data are expressed as the number of Ki-67-positive cells/total number of tumor cells/field. *p<0.05. (E) A representative photograph of H&E staining of a tumor from the control and IMD-0560-treated groups *in vivo* (magnification 400x). Bar=100 μm. (F) A representative photograph of MMP-9 staining of a tumor from the control and IMD-0560-treated groups *in vivo*. MMP-9-positive SCCVII cells were clearly identifiable based on brown staining (magnification 400x). Bar=100 μm. (G) The number of MMP-9-positive cells was counted in 5 randomly selected fields of each tumor specimen. The data are expressed as the percentage of MMP-9-positive cells/total number of tumor cells/field. *p<0.01.

Histological analysis revealed of the mandibular bone, including irregularities in the bone surface and an area of osteoclast occupation at the tumor/bone interface, in the control group and some of the animals in the 3 mg/kg IMD-0560-treated group. In the 5 mg/kg IMD-0560-treated group, tumors were detected far from mandibular bone, but they were not attached to the bone surface, and the mandibular surface was smooth (Figure 6A, upper panels). Many osteoclasts were easily identified in the control group, and osteoclasts were observed along the resorbed lacunae in the 3 mg/kg IMD-0560-treated group (Figures 6A, middle panels and 6B). Few osteoclasts were detected in the 5 mg/kg IMD-0560-treated group (Figures 6A, middle panels and 6B). The SCCVII cells and the osteoblastic cells located at the interface between the bone and the SCCVII cells strongly expressed RANKL in the control group (Figure 6A, bottom panels). RANKL expression in both the SCCVII cells and the osteoblastic cells was reduced in the 3 mg/kg IMD-0560-treated group compared to the control group. No RANKL-positive cells were detected near the bone surface in the 5 mg/kg IMD-0560-treated group (Figure 6A, bottom panels).

The number of Ki-67-positive cells in the tumors was similar between the 3 mg/kg IMD-0560-treated group and the control group. Treatment with the higher dose of IMD-0560 strongly reduced the number of Ki-67-positive cells (Figures 6C and 6D). The tumor tissue was nearly intact in the control group, whereas small necrotic foci were detected in the 3 mg/kg IMD-0560-treated group. The tumor tissue in the 5 mg/kg IMD-0560-treated group was largely replaced by necrotic and granulated tissue (Figure 6E). Treatment with 3 mg/kg IMD-0560 failed to reduce the number of MMP-9-expressing tumor cells, but 5 mg/kg IMD-0560 greatly reduced the number of MMP-9-expressing tumor cells (Figures 6F and 6G).

## DISCUSSION

Aberrant NF-κB activity is implicated in many cancers, including OSCC, and contributes to the acquisition of malignant characteristics, such as increased cell invasion, cell survival, chemoresistance, and angiogenesis [13-15]. The therapeutic potential of IKKβ-dependent NF-κB pathway disruption in cancers has been extensively studied using biochemical approaches [19, 20]. The dominant negative form of IKKβ, which lacks conserved lysine residue that is necessary for its catalytic activity, or non-degradable IκBα, which lacks its serine phosphorylation sites, reduced inflammatory cytokine expression and tumor growth *in vitro* and *in vivo* [21, 25, 26], suggesting that IKKβ inhibition represents a very promising therapeutic strategy for cancer. To date, several pharmacological inhibitors of NF-κB have been developed, and these inhibitors have been demonstrated to effectively reduce cancer cell growth and invasion [13, 14]. Additionally, we have shown that a selective inhibitor of NF-κB, NBD peptide, inhibited bone invasion by SCCVII cells via the suppression of osteoclastic bone resorption, increased apoptosis, and suppressed the proliferation of SCCVII cells [21]. However, it is very difficult to apply this laboratory reagent to clinical use.

IMD-0560 and its prodrug IMD-2560 are recently synthesized agents that inhibit IKKβ activity under inflammatory stimuli and the subsequent production of cytokines [19-24]. IMD-0560 potently inhibited TNFα-induced IL-6 production in human fibroblast-like synoviocytes derived from rheumatoid arthritis patients (HFLS-RA) cells and TNFα-induced NF-κB activation in HEK293T cells without causing cell toxicity, although its potency greatly varies depending on the cell type, incubation time and other conditions [22-24]. In this study, we used IMD-0560 from 1 to 10 μM to inhibit MMP-9 activity or p65 phosphorylation. The *in vivo* efficiency of IMD-0560 has been demonstrated, as intraperitoneal injection of DBA/1J mice with 1 or 3 mg/kg IMD-0560 every 48 hours beginning at the first immunization resulted in a significant and dose-dependent reduction in the incidence, severity, and pathological abnormalities of collagen-induced arthritis [22]. Moreover, IMD-0560 significantly improved cardiac function in a rat myocardial ischemia model [23]. Furthermore, a phase I study has been completed using IMD-2560, which is reportedly under assessment for rheumatoid arthritis [20]. These results led us to investigate whether IMD-0560 inhibits bone invasion by OSCC using an animal model.

Bone invasion by OSCC consists of a complex, multistep cascade of events, and these events are regulated by a wide variety of molecules that contribute to cell invasion of jaw bones, including the degradation of the extracellular matrix of gingiva and osteoclastogenesis [4,5]. Several *in vitro* and animal experiments using OSCC cells have shown that tumor cells produce several cytokines, including interleukin-6 (IL-6), IL-11, TNFα and parathyroid hormone-related protein (PTHrP). These cytokines regulate the expression of RANKL and osteoprotegerin [9,10]. In this model, RANKL expression was observed in both SCCVII cells and osteoblastic cells that faced the bone surface. IMD-0560 suppressed RANKL expression in both SCCVII cells and osteoblastic cells might be due to induce SCCVII apoptosis or to lead less production of cytokines, which induce RANKL.

MMPs, such as MMP-1, -2, -9 and -14, are involved in the invasive properties of OSCC via the degradation of the extracellular matrix [27], and MMP expression is regulated by NF-κB [28]. A previous report showed that the NF-κB binding site in the MMP-9 promoter region in human fibroblasts is necessary for the inflammatory cytokine-mediated induction of MMP-9 expression, and we previously reported that the expression and activity of MMP-9, but not MMP-2, are regulated by NF-κB [29]. TNFα stimulated the invasion of HSC-2 and SCCVII cells, but not Ca_9-22_ cells, by increasing MMP-9 mRNA and protein expression and activity. Ca_9-22_ cells display limited invasion potential and MMP-9 protein expression [30]. IMD-0560 treatment suppressed MMP-9 activity by inhibiting MMP-9 mRNA expression, suggesting that IMD-0560 suppressed MMP-9 activity at the transcriptional level. In support of this conclusion, injection of IMD-0560 reduced the number of MMP-9-positive SCCVII cells *in vivo.* IMD-0560 treatment also suppressed MMP-2 production in SCCVII and HSC-2 cells. Although IMD-0560 did not notably affect cell viability, it might slightly damage these cells.

The most important aspect of this study compared with former experiments that used other pharmacological NF-κB inhibitors is that the inhibitor used in this study effectively prevented bone invasion upon initiation of 5 mg/kg IMD-0560 injection at a relatively advanced stage of tumor growth. In the previous study and this study, we initiated the injection of NBD peptide or IMD-0560 immediately after tumor implantation. Although injection of NBD peptide or IMD-0560 sufficiently inhibited bone invasion by SCCVII cells in these experiments, patients hardly recognize any sign of ill-health at the early stage of OSCC in clinical practice. Thus, we used a “late treatment model” in which IMD-0560 injection was initiated after confirming that the tumor had become large. Treatment with 5 mg/kg IMD-0560 reduced the tumor size by up to half, but zygoma destruction was sufficiently blocked. Histological analysis revealed the tumor, but it appeared necrotic and did not reach the bone surface. Furthermore, cell proliferation and RANKL and MMP-9 expression in SCCVII cells were strongly inhibited by treatment with 5 mg/kg IMD-0560. So far, severe side effects of IMD treatment have not been reported during the phase I clinical trial [20]. Since it is generally agreed that patients with mandibular invasion should be treated surgically, these results indicate that a patient's burden might be mitigable, if the area of a surgical resection could be smaller after IMD-0560 treatment. Although we alternatively initiated 5 mg/kg IMD-0560 injection 3 weeks after SCCVII injection, when the tumor cells have invaded the bone, this treatment failed to prevent death because tumor cell growth was too rapid to control in this experiment (data not shown). It is likely that other pathways independent of the IKKβ-NF-κB pathway were activated before administering IMD-0560.

In conclusion, it is noteworthy that NF-κB activity increased in association with the enhancement of the bone invasive potential of OSCC. Selective inhibition of NF-κB activation using the novel IKKβ inhibitor IMD-0560 reduced bone invasion in an animal model. In the case of mandibular bone invasion, it is possible to locally treat cancer with IMD-0560 to avoid any unexpected severe side effects because OSCC cells invade from the cortical bone and because the tumor and defect are at the surface of the body. Previous reports have demonstrated the safety of IMD-0560 administration [22-24]. Similar results demonstrated that IMD-0560 treatment does not affect the proliferation of cells in the basal and parabasal layers of the tongue (Supplementary Figure S5), suggesting that IMD-0560 treatment specifically affects tumor cells. Thus, upon the establishment of a suitable protocol for IMD-0560 treatment, our results will provide a new potential compound for the treatment of bone invasion by OSCC.

## MATERIALS AND METHODS

All protocols for the present experiments were reviewed and approved by the Council on Animal Care at Kyushu Dental University (Approval number 13-006).

### Reagents

The anti-p65 (sc-109), anti-IκBα (sc-371), anti-RANKL (sc-7628) and anti-rabbit IgG (sc-2004) antibodies were obtained from Santa Cruz Biotechnology (Santa Cruz, CA, USA). The anti-phosphorylated p65 (Serine 536) (#3031) and anti-mouse IgG antibodies were obtained from Cell Signaling (Beverly, MA, USA) and Amersham Bioscience (Piscataway, NJ, USA), respectively. The anti-rat matrix metalloproteinase-9 (MMP-9) antibody (AB19016) was obtained from Millipore (Billerica, MA, USA). The anti-β-actin antibody (AC-15) was purchased from Sigma-Aldrich (St. Louis, MO, USA).

### Cell culture

The human OSCC cell lines used in our study were HSC-2 and Ca_9-22_, which were purchased from the Japanese Collection of Research Bioresources (JCRB) Cell Bank (Tokyo, Japan). SCCVII cells, derived from a mouse OSCC cell line, were cultured in Dulbecco's modified Eagle's medium (DMEM) containing 5% fetal bovine serum (FBS), 100 units/ml penicillin, and 100 mg/ml streptomycin (Sigma-Aldrich) in a humidified atmosphere containing 5% CO_2_ at 37°C.

### Western blot

For immunoblotting, whole-cell lysates were resolved on SDS polyacrylamide gels, transferred to PVDF membranes and incubated at 4°C overnight in antibodies diluted to 1:1,000 in a 5% dry milk or bovine serum albumin (BSA) solution containing 0.01% azide in TTBS (10 mM Tris-HCl, 50 mM NaCl and 0.25% Tween-20), followed by incubation in a horseradish peroxidase-conjugated secondary antibody. The immunoreactive proteins were visualized using Enhanced ChemiLuminescence (Millipore).

### Immunofluorescence microscopy

Immunofluorescence analysis was performed as previously described [21]. Briefly, the OSCC cells were pretreated with various concentrations of IMD-0560 for 120 min and then treated with TNFα (10 ng/ml) for 30 min. The cells were fixed with 3.7% formaldehyde and 0.2% glutaraldehyde, blocked with 5% skim milk in PBS and incubated in an anti-human p65 polyclonal antibody (1:100) overnight at 4°C. Then, the cells were incubated in Alexa Fluor 430-conjugated anti-rabbit IgG (1:10,000, Invitrogen, Carlsbad, CA, USA) for 90 min at 37°C. The subcellular localization of Alexa Fluor 430-labeled p65 was determined via fluorescence microscopy (Biorevo, BZ-9000, Keyence, Osaka, Japan). To visualize the nuclei, the cells were stained with DAPI.

### Luciferase assay

The luciferase assay was performed as previously described [17,19,21]. The SCCVII cells were transfected with the PBIIx-luciferase reporter using GeneJuice transfection reagent (Merck, Darmstadt, Germany) according to the manufacturer's instructions. The luciferase activity was measured using a dual luciferase reporter assay system (Promega, Madison, WI, USA).

### Cell invasion assay

Cell migration was assessed in a modified Boyden chamber containing a gelatin-coated porous membrane. IMD-0560 was placed in the upper chamber 2 hrs prior to TNFα treatment, and TNFα was placed in the lower chamber at 10 ng/ml; then, the chamber was incubated for 24 hrs. Next, the cells attached to the upper surface of the membrane were scraped off, and the cells that migrated to the lower surface were fixed and stained with DAPI.

### Zymography

The serum-free conditioned medium was collected from a confluent culture of OSCC cells incubated in the presence of reagents for 24 hrs. The conditioned medium was resolved via 10% SDS-PAGE in the presence of 1 mg/ml gelatin. The resulting gel was washed in 10 mM Tris (pH 8.0) containing 2.5% Triton X-100, followed by incubation for 16 hrs in a reaction buffer (50 mM Tris, pH 8.0, 0.5 mM CaCl_2_, and 10^−6^ M ZnCl_2_) at 37°C. After staining with Coomassie brilliant blue R-250, the gelatinases were identified as clear bands of lysed gelatin against a blue background.

### Real-time reverse transcription polymerase chain reaction (RT-PCR)

Total RNA from the OSCC cells was prepared using TRIzol reagent (Invitrogen) and then reverse-transcribed into cDNA. Real time RT-PCR was performed as previously described [31]. The primer sequences were shown in Supplementary Table 1.

### Animal model of bone invasion

Sixty male C3H/HeN mice, which were obtained from CLEA (Tokyo, Japan) weighing approximately 20 g at 8 to 10 weeks of age were used to establish a model of mandible invasion by OSCC cells [32, 33]. The animal experiments were conducted according to the guidelines for the treatment of experimental animals of Kyushu Dental University. The mice were randomly separated into six groups with similar average body weights. They were anesthetized using ether, and 0.1 ml of SCCVII (1.0 × 10^5^/ml) in DMEM was injected into the left masseter region. One week (early treatment: E) or 2 weeks (late treatment: L) after injection, the mice were injected between the left masseter region and the surface of the left mandibular bone with vehicle (50 μl of carboxymethyl-cellulose: CMC/mouse, n=10) or IMD-0560 (3 or 5 mg/kg/50 μl of CMC/mouse, n=10), 3 times per week for a total of 3 weeks (E) or 2 weeks (L). The tumor sizes were assessed using calipers, and the tumor volume was calculated using the following formula: width^2^ × length × 0.52 [34]. At the end of week 3, all surviving mice were euthanized, and the heads of the mice were fixed in 3.7% formaldehyde (Wako Pure Chemical Industries, Ltd., Osaka, Japan).

Three-dimensional (3-D) reconstructed images of the heads were obtained via microfocal computed tomography (μ-CT) (ScanXmate-E090, Comscan, Kanagawa, Japan) as previously described (21). The extent of zygoma destruction was scored using the reconstructed μ-CT images as follows: 0: normal; 1: asymmetric; 2: displaying a hairline fracture; 3: zygoma completely separated; and 4: destruction of more than 1/3 of the zygoma. The images were evaluated in a blinded manner by 8 researchers [21].

Formaldehyde-fixed paraffin-embedded sections at a thickness of 5 μm were generated posterior of the lower third molar and were stained with hematoxylin & eosin (H&E) and tartrate-resistant acid phosphatase (TRAP). Some specimens were processed for immunohistochemical staining with anti-RANKL and anti-MMP-9 antibodies (1:100) using an Imm PRESS^TM^ Reagent kit (Vector Laboratories, Burlingame, CA, USA) and Histo Fine Simple Stain MAX-PO (R) (Nichirei, Tokyo, Japan), respectively. A non-immunoreactive immunoglobulin G antibody was used as a negative control for immunohistochemistry. All samples were observed under a microscope.

All incubations were performed at room temperature. For each specimen, 5 tumor fields were randomly selected, and the number of TRAP-positive multinucleated cells (TRAP^+^ MNCs) was counted. The data are expressed as the numbers of TRAP^+^ MNCs/bone surface (mm^2^)/section.

### Proliferation assay

The 5-μm sections were stained with an anti-mouse Ki-67 monoclonal antibody (1:100, ab15580, Abcam, Cambridge, UK) using the immunoperoxidase technique (Dako LSAB2 System; HRP, Dako, Glostrup, Denmark). Five tumor fields for each specimen were randomly selected, and the number of Ki-67-positive cells was counted. The data are expressed as the numbers of Ki-67-positive cells/mm^2^ tumor area.

### Data analysis

The comparisons were performed using an unpaired Student's *t*-test. The data are expressed as the mean ± SD; p values <0.05 were considered to be significant. For the *in vivo* experiments, the comparisons were performed via factorial ANOVA. When significant F values were detected, Fisher's PLSD post hoc test was performed for between-group comparison. The data are expressed as the mean ± SD; p values < 0.05 were considered to be significant.

## SUPPLEMENTARY MATERIAL, FIGURES AND TABLE


