# Supplementary material for: The novel IκB kinase β inhibitor IMD-0560 prevents bone invasion by oral squamous cell carcinoma
Source: Oncotarget. 2014 Oct 28;5(23):12317–30. doi: 10.18632/oncotarget.2640 (PMC4322973; doi:10.18632/oncotarget.2640)
Supplement: Supplementary file 1 [file oncotarget-05-12317-s001.pdf]

The novel IκB kinase β inhibitor IMD-0560 prevents bone invasion by oral squamous cell carcinoma

Supplementary Material

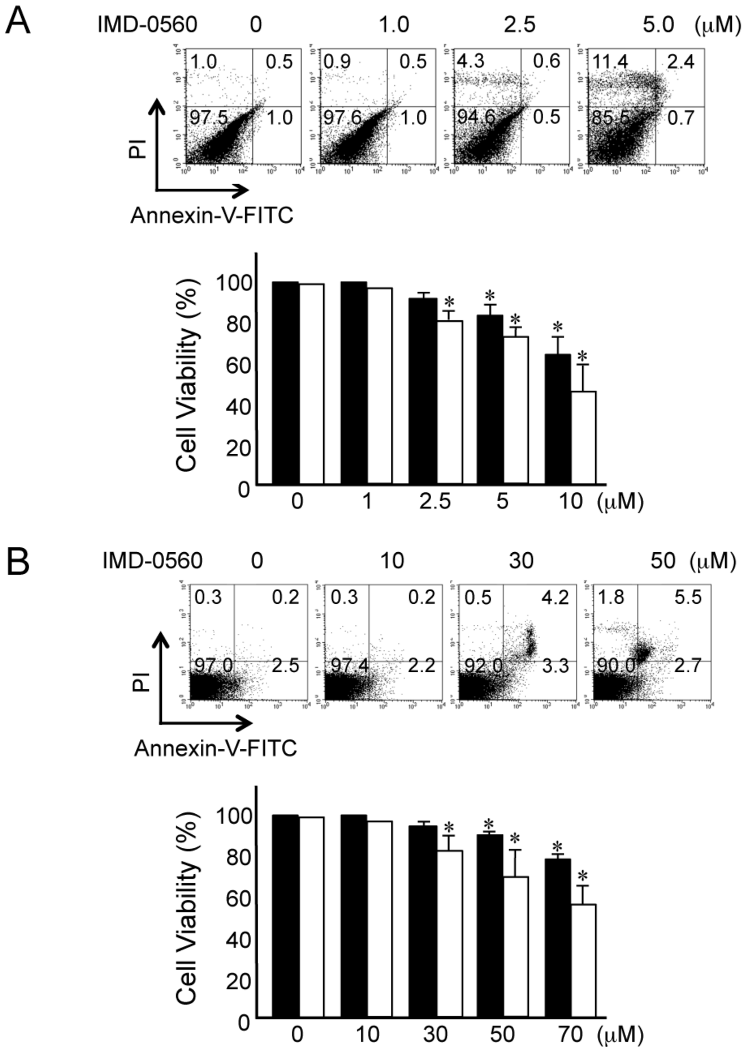

Supplementary Figure S1. IMD-0560 induces apoptosis in SCCVII cells *in vitro*. SCCVII (A) and HSC-2 (B) cells were cultured in the presence of various concentrations of IMD-0560 treatment for 24 (closed column) or 48 (open column) hrs *in vitro*. The cells were stained with FITC-conjugated Annexin V and PI and were analyzed via two-color flow cytometry. The data are representative of three independent analyses for 24 hrs. The living cells (Annexin V^-/PI^- cells) were counted via flow cytometry. Similar results were obtained in three independent experiments. The data are expressed as the mean ± SD (n=3). *p<0.01.

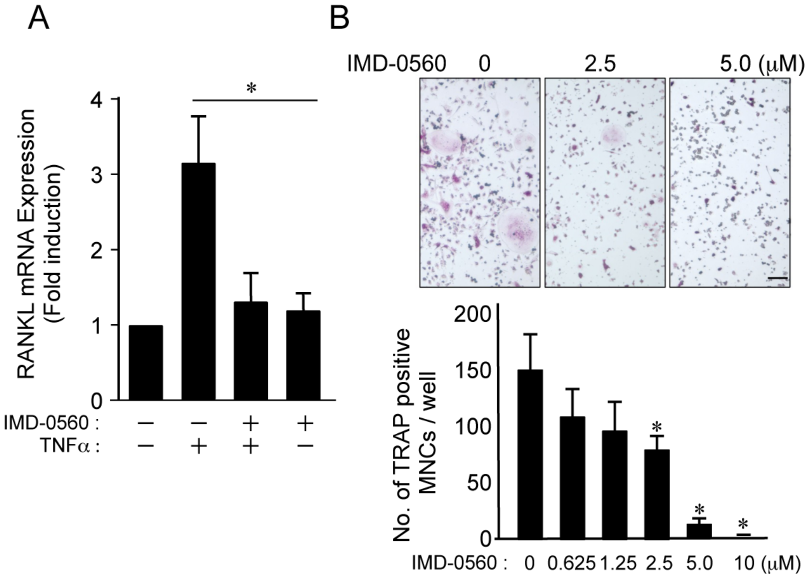

Supplementary Figure S2. IMD-0560 inhibits RANKL expression in osteoblasts and SCCVII cells and RANKL-induced osteoclastogenesis. (A) Primary osteoblasts were cultured in the presence or absence of IMD-0560 (10 μM) with or without TNFα (10 ng/ml) treatment for 48 hrs. Total RNA was isolated from osteoblasts, and the expression levels of *RANKL* relative to those of *β-actin* were measured via real-time PCR analysis. Similar results were obtained in three independent experiments. The data are expressed as the mean ± SD (n=3). *$p<0.01$. (B) Bone marrow cells from the tibia were cultured in the presence of RANKL and M-CSF together with various concentrations of IMD-0560. The cells were fixed and stained for TRAP (magnification 100x) Bar=100 mm. The TRAP^{+} MNCs were counted as osteoclasts. The data are presented as the numbers of TRAP^{+} MNCs per culture well. Similar results were obtained in three independent experiments. The data are expressed as the mean ± SD (n=3). *$p<0.01$.

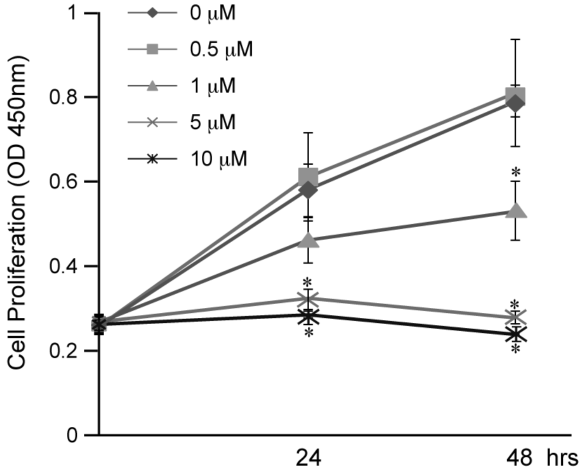

Supplementary Figure S3. IMD-0560 suppresses the proliferation of SCCVII cells *in vitro*. SCCVII cells were cultured in the presence of various concentrations of IMD-0560 for 24 or 48 hrs *in vitro*. The proliferation of SCCVII cells was assessed using a Cell Counting Kit-8 (Dojin, Kumamoto, Japan), according to the manufacturer's protocol. Similar results were obtained in three independent experiments. The data are expressed as the mean ± SD (n=3). *$p<0.01$.

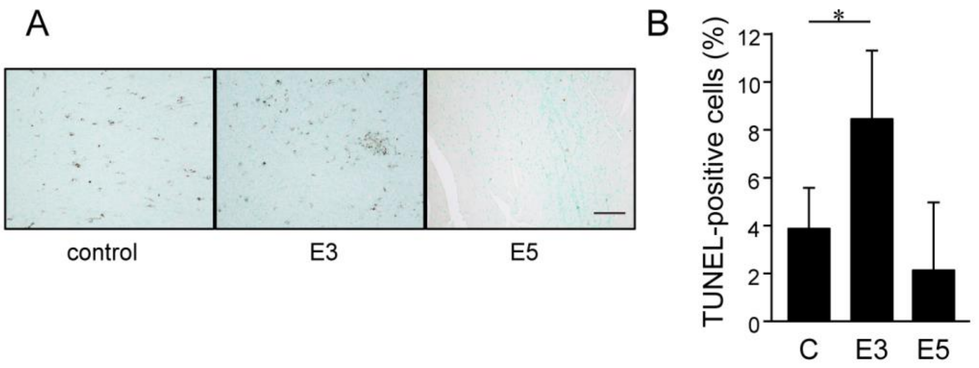

Supplemental Figure S4. IMD-0560 induces apoptosis in SCCVII cells *in vivo*. (A) A representative photograph of TUNEL staining (ApopTag Plus *in situ* Apoptosis Detection kit Oncor, Gaitherburg, MD) in SCCVII of control and IMD-0560 at 3 or 5 mg/kg for 3 weeks *in vivo*. TUNEL-positive apoptotic SCCVII cells are clealy identifiable by brown staining (magnification 200x). Carcinoma cells were nearly absent from the site of cell injection in the 5 mg/kg IMD-0560-treated group. Bar = 100 μm. (B) In each specimen, 5 tumor fields were randomly selected, and the number of TUNEL-positive cells was counted. The data are expressed as the mean ± SD of the number of TUNEL-positive cells/total number of tumor cells/field (n=10). **p*<0.05. Similar results were obtained in three independent experiments.

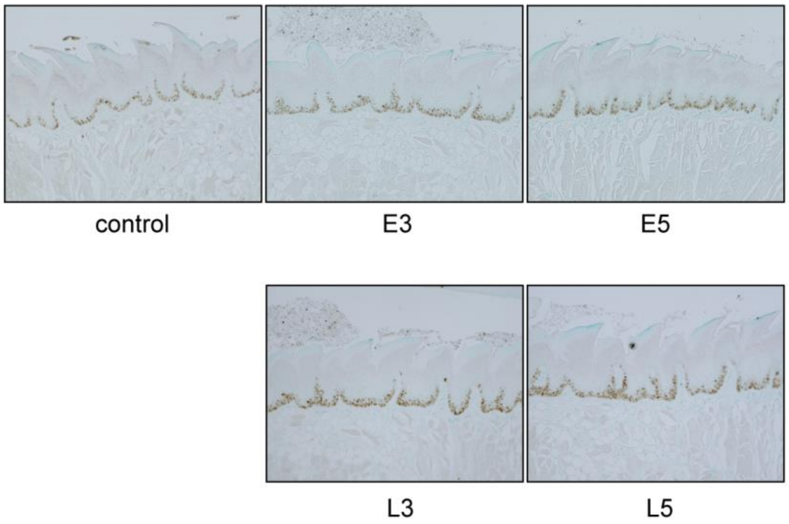

Supplemental Figure S5. IMD-0560 did not affect the proliferation of cells in the basal and parabasal layers of the tongue. Tumor-bearing mice were pretreated with vehicle (control) (50 μl of CMC, n=10) or IMD-0560 (3 or 5 mg/kg in 50 μl of CMC, n=10 each) 3 times per week for 2 (late treatment: L) or 3 (early treatment: E) weeks. At the end of the third week of treatment, all surviving mice were euthanized, and the heads of the mice were fixed in 3.7% formaldehyde. A representative photograph of Ki-67 staining of the cells in the basal and parabasal layers of the tongue treated with IMD-0560 is shown. Ki-67-positive cells in the basal and parabasal layers of the tongue were clearly identifiable based on brown staining (magnification 200x). Mice treated with CMC alone served as controls. C: control, E3: mice treated with IMD-0560 at 3 mg/kg for 3 weeks, E5: mice treated with IMD-0560 at 5 mg/kg for 3 weeks, L3: mice treated with IMD-0560 at 3 mg/kg for 2 weeks, L5: mice treated with IMD-0560 at 5 mg/kg for 2 weeks. Bar=100 μm.

Supplementary Table 1. PCR primers used for study.

species	target mRNA	reverse	reverse
human	β-actin	5'-ccaaccgcgagaagatga-3'	5'-ccagaggcgtacagggatag-3'
human	MMP-9	5'-acctcgaactttgacagcgac-3'	5'-gaggaatgatctaagcccagc-3'
mouse	β-actin	5'-aaggccaaccgtgaaaagat-3'	5'-gtggtacgaccagaggcatac-3'
mouse	RANKL	5'-agccatttgcacacctcac-3'	5'-cgtggtaccaagaggacagagt-3'
mouse	MMP-9	5'-cttctggcgtgtgagtttcc-3'	5'-actgcacggttgaagcaaaga-3'
